# The Use of Spatial Data Infrastructure in Environmental Management:an Example from the Spatial Planning Practice in Poland

**DOI:** 10.1007/s00267-016-0732-0

**Published:** 2016-06-29

**Authors:** Agnieszka Zwirowicz-Rutkowska, Anna Michalik

**Affiliations:** 1 Faculty of Geodesy, Geospatial and Civil Engineering, University of Warmia and Mazury, ul. Oczapowskiego 2, 10-719 Olsztyn, Poland; 2 Urbaneo, Urban Design Studio, ul. Tęczowy Las 4/48, 10-687 Bartąg, Olsztyn, Poland

**Keywords:** Spatial data infrastructure (SDI) usability, Urban planner, Spatial plan, Decision making, Environmental indicator, Nature conservation

## Abstract

Today’s technology plays a crucial role in the effective use of environmental information. This includes geographic information systems and infrastructures. The purpose of this research is to identify the way in which the Polish spatial data infrastructure (PSDI) supports policies and activities that may have an impact on the environment in relation to one group of users, namely urban planners, and their tasks concerning environmental management. The study is based on a survey conducted in July and August, 2014. Moreover, the authors’ expert knowledge gained through urban development practice and the analysis of the environmental conservation regulations and spatial planning in Poland has been used to define the scope of environmental management in both spatial planning studies and spatial data sources. The research included assessment of data availability, infrastructure usability, and its impact on decision-making process. The results showed that the PSDI is valuable because it allows for the acquisition of data on environmental monitoring, agricultural and aquaculture facilities. It also has a positive impact on decision-making processes and improves numerous planners’ activities concerning both the inclusion of environmental indicators in spatial plans and the support of nature conservation and environmental management in the process of working on future land use. However, even though the infrastructure solves certain problems with data accessibility, further improvements might be proposed. The importance of the SDI in environmental management is noticeable and could be considered from many standpoints: Data, communities engaged in policy or decision-making concerning environmental issues, and data providers.

## Introduction

One of the factors influencing the task and process flows related to environmental management and the quality of services in the area of environmental management is the wide range of resources involving data and technological solutions. The International Telecommunication Union (ITU) (2008) summarizes the role of information and communication technologies (ICTs) in the e-environment, which includes, among other things, the collection and management of environmental information as well as understanding the impact that humans exert on the environment. The issue of facilitating access to biodiversity information in relation to users’ needs in the area of managing the environment is raised by Davis et al. (2014). Many other authors (e.g., Wilcox 1990; Longley et al. 2005; Masser 2007; Craglia and Campagna 2010) emphasize the benefits of spatially oriented ICT, i.e., the geographic information system (GIS) and the spatial data infrastructure (SDI), in many sectors and various applications. A review of the literature on environmental management shows numerous successful applications of the GIS (Gonzalez et al. 2011; Gonzalez Del Campo 2012; Emili and Greene 2013; Gebbert and Pebesma 2014; Inanloo and Tansel 2015). Tuchyna (2006) shares his experiences involving the process of SDI development within the environmental sector, and Latre et al. (2013) present SDI support for environmental e-government services.

SDI on different levels of government may become a powerful tool in various applications, including environmental management. On the European level, the role of Infrastructure for Spatial Information in the European Community (INSPIRE) in assisting policy-making in relation to policies and activities that may have a direct or indirect impact on the environment is well articulated (European Parliament and the Council 2007). Still, the successful application and use of SDI products and services (Nedović-Budić et al. 2008) relies on the fulfillment of the needs and expectations of the users. An overview of literature shows that SDI assessment from the viewpoint of the users in general and among different thematic groups of beneficiaries has not been yet thoroughly investigated, although the results of research on the case studies of SDI use have been demonstrated (e.g., Askew et al. 2005; Vandenbrucke et al. 2013).

The aim of the paper is to identify and describe the Polish spatial data infrastructure support for policies and activities that may have an impact on the environment in relation to one group of users, i.e., urban planners, and their activities concerning environmental management. A further objective is to analyze and discuss the inclusion of environmental issues in the process of preparing local plans, i.e., institutions, data sources, and planner decisions about future land use.

The contribution of the paper is an ex-post evaluation from the users’ standpoint as well as the presentation of certain initial findings regarding the usefulness and usability of selected components of the Polish Spatial Data Infrastructure (i.e., the main access point and its applications, spatial data and services) in realization of tasks related to the environmental impact assessment performed by urban planning professionals. Moreover, the paper includes an analysis and discussion of the role and importance of national, regional, and thematic access points to metadata and spatial data services in the area of environmental management, as well as an assessment of the coordination of the data contributing and providing processes used by institutions within the infrastructure from the perspective of the SDI users. The results may serve as the basis for defining improvements to SDI development in order to better suit the needs of the users in terms of both data access and analysis.

## The Polish Spatial Data Infrastructure

**Fig. 1:**
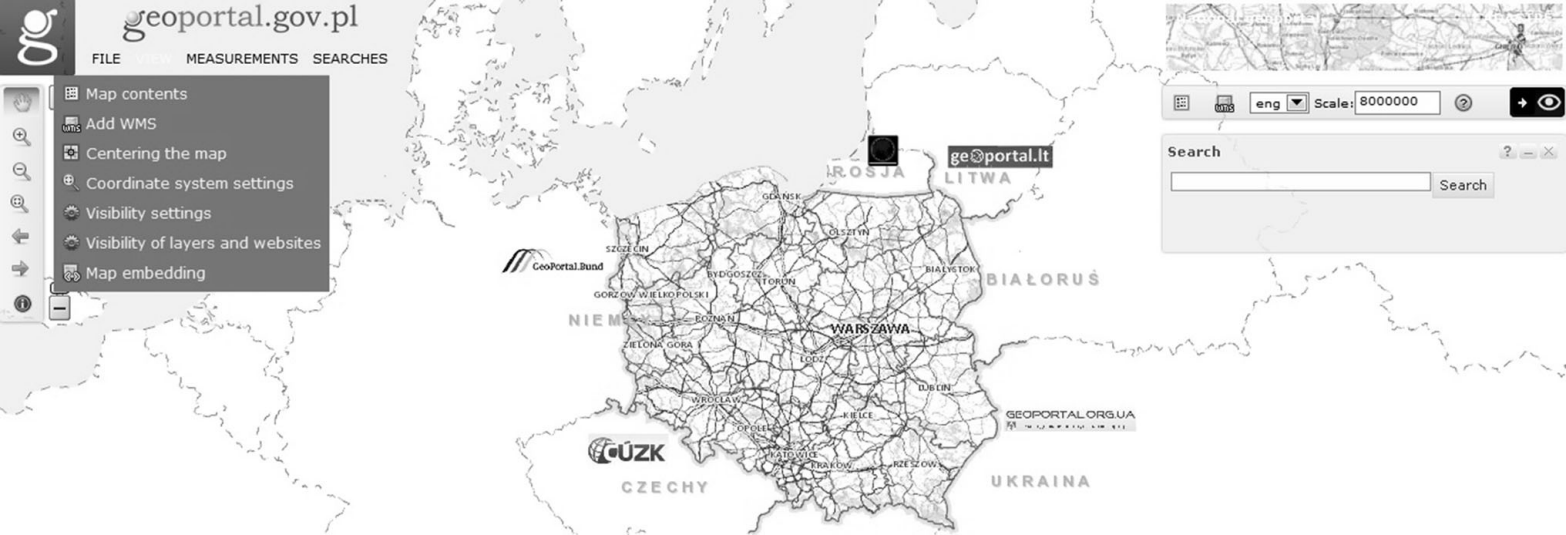
Map viewer in the Polish geoportal

In Poland, the spatial information infrastructure was established by the Act on Spatial Information Infrastructure (Polish Parliament 2010). The Act specifies the principles of operation of the SDI and introduces mechanisms which enable interoperability of spatial data, metadata, and network services. The national access point called Geoportal 2 offers applications, such as a national geoportal, a geoportal for INSPIRE, a metadata editor and validator, mobile applications, and a statistics module. Table 1 illustrates the main features and functions of the map viewer in the national geoportal (Fig. 1).

**Table 1:** Map viewer functions (Zwirowicz-Rutkowska 2016)

No.	Function
1.	Set map-scale command
2.	Pan command
3.	Zoom-in/out command
4.	Info about feature command
5a.	File menu—link to map composition
5b.	File menu—user’s maps
6a.	View menu—map content
6b.	View menu—add maps
6c.	View menu—center map
6d.	View menu—set coordinate system
6e.	View menu—set toolbar visibility
6f.	View menu—set data layer and service visibility
6g.	View menu—generate HTML code
7a.	Measurement menu—measure area
7b.	Measurement menu—measure distance
8a.	Search menu—search for addresses and features
8b.	Search menu—search for cadastral parcels
8c.	Search menu—metadata

**Fig. 2:**
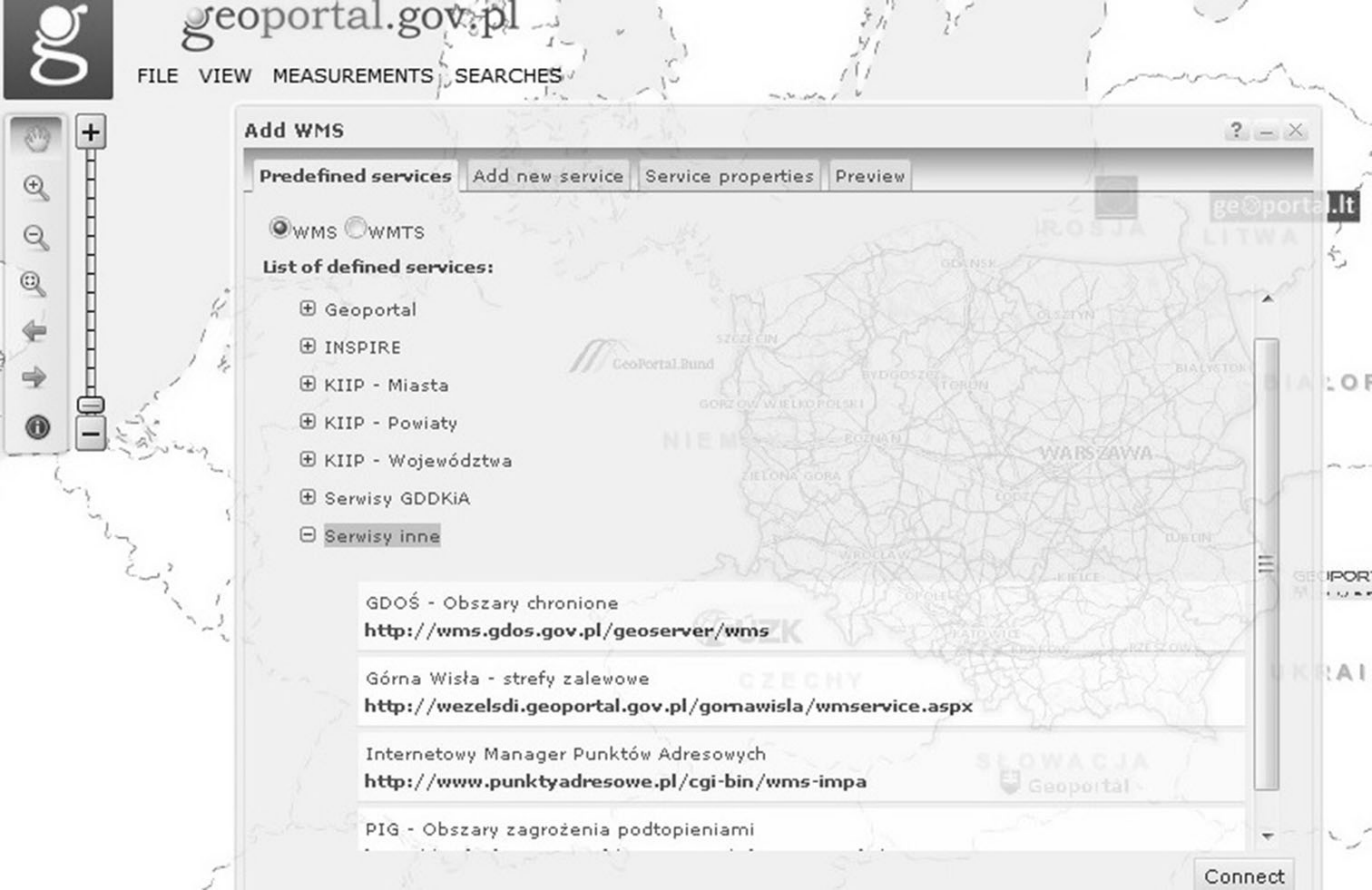
The Polish access point as the broker

**Fig. 3:**
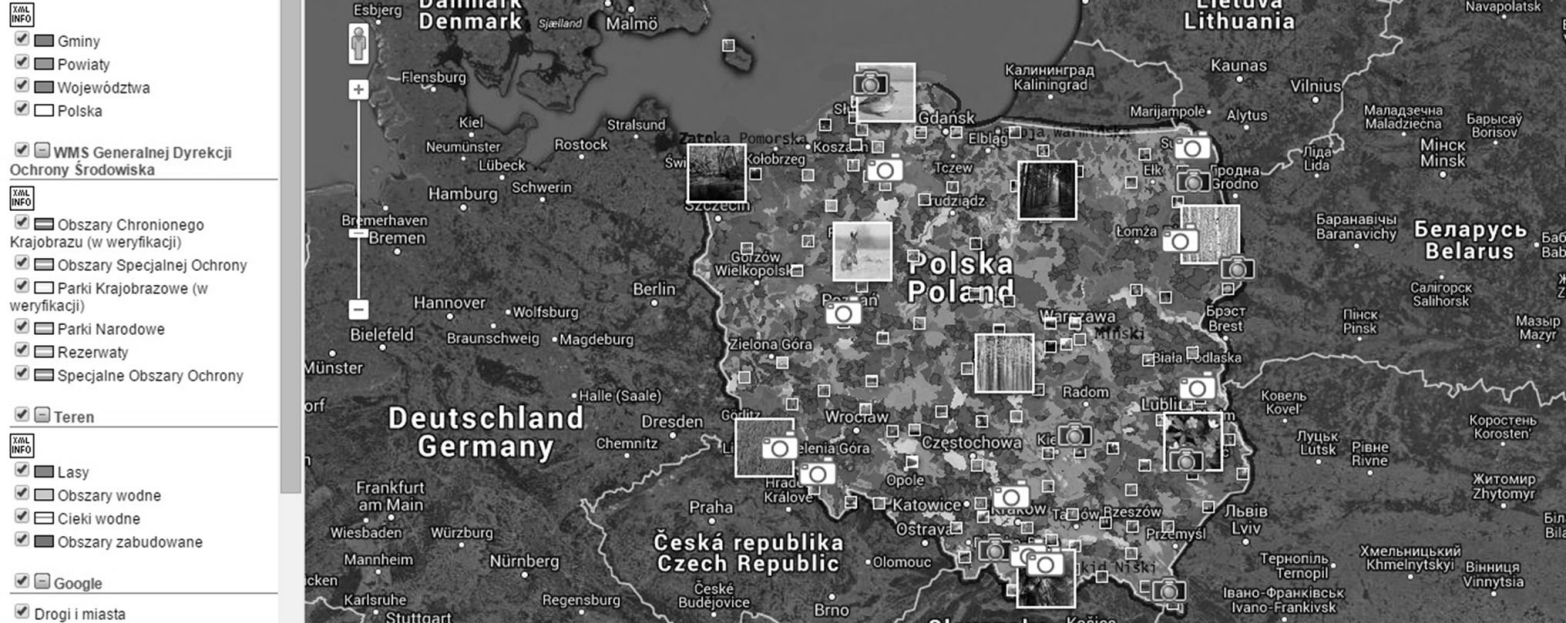
The environmental geoportal with conservation areas (http://mapa.ekoportal.pl/. Accessed January 2015)

**Fig. 4:**
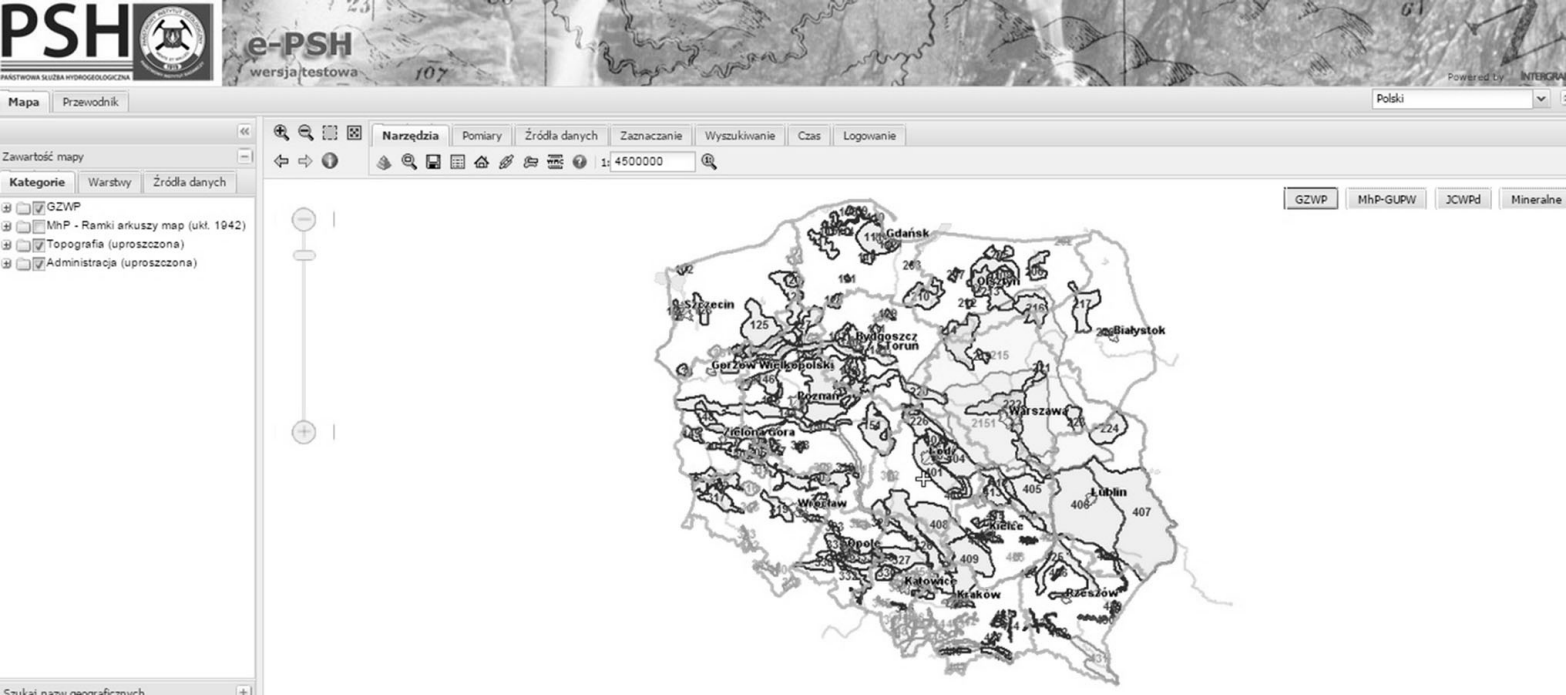
The environmental geoportal with underground bodies of water (http://epsh.pgi.gov.pl/epsh/. Accessed January 2015)

The possibility of incorporating third party services into the Polish spatial data infrastructure is undoubtedly one of the factors ensuring the usefulness of the national access point. The Polish spatial data infrastructure (Zwirowicz-Rutkowska 2016) is also developed on national, regional, and local levels through different initiatives and by many institutions and government departments. The national geoportal integrates certain thematic, regional, and local geoportals (Fig. 2). Nonetheless, there are no formal obligations to include the outputs of these initiatives (i.e., geoportals) in the national access point. Figs. 3, 4 show example geoportals which are not included in the national access point.

## Methods

The study is based on a survey conducted among urban planners in July and August, 2014. The survey instrument consisted of closed-ended questions referring to some data on the professional activities of the urban planners (the firm’s size, the scope of the activities, number of employees using the PSDI and different geoinformation web sites) and the usefulness of the national access point (a geoportal called Geoportal 2 and its data content) and PSDI support for decision making. For quantitative research, a 0–10 scale was proposed. A total of 63 questionnaires were mailed to companies specializing in local level planning. Data about spatial planners were obtained from the web service of the National Chamber of Urban Planners. A list of spatial planners included all four district societies. For survey purpose, 15 firms were selected from the Western District Urban Planners Society with its seat in Wrocław, 18 firms from the Central District Urban Planners Society with its seat in Warszawa, 20 firms from the North District Urban Planners Society with its seat in Gdańsk, and 10 firms from the South District Urban Planners Society with its seat in Katowice. The achieved completion rate was 44.4 %. The respondents were mainly the representatives of the small or midsize firms specializing in factor and land use trends analyses, production of a comparative study of variants and local spatial plans, and field studies. The reported user group of the PSDI Geoportal 2 and other geoinformation web sites in their organizations was “some” or “one or none.”

Furthermore, the expert knowledge gained through urban development practice and the analysis of the regulations on environmental conservation and spatial planning in Poland has been used to define the scope of environmental management in both spatial planning studies and spatial data sources. To assess the degree of usage of the Polish Spatial Data Infrastructure in the area of urban tasks concerning environmental issues, quantitative and qualitative studies were conducted.

## The Structure of Spatial Planning Documents in Poland

The scope of urban tasks depends on the type of spatial planning documentation. According to the Act on Spatial Planning and Land Development (Polish Parliament 2015), the national spatial strategy, which defines determinants as well as the objectives and goals of sustainable development along with actions aimed at supporting their realization is the predominant document. Lower-order studies include regional spatial development plans. Spatial policy on the province level is defined through the studies of the conditions and directions of spatial management. The studies indicate rules governing the local spatial management and are binding for local authorities in the process of preparing local plans. Local plans depict the planned land use as well as the conditions of spatial management and building development. Public investments are allocated on the basis of either local plans or, if there are no legal plans, decisions on location conditions for public investments. Any change in the land use in the aforementioned case requires issuing a decision on building conditions. Hierarchy and dependence of the spatial planning documents are the key feature of the spatial planning system in Poland.

## A Case Study of Local Plans

The process of preparing a spatial plan is rather complex and depends on the collaboration between many different institutions and authorities. An overview of such a process and its participants is presented in Sect. 5.1. Particular emphasis is placed on the role of the planner and their tasks as well as different institutions connected with environmental management involved in the urban studies. “Local plans and environmental resource management” section portrays the results of the qualitative analysis of the relations between local plans and environmental resource management. Urban planners are required to include many different environmental indicators in the process of planning future land use. Moreover, the planned land use may also have an impact on the environment. In “Data sources in the National Spatial Data Infrastructure” Sect, certain quantitative assessment results concerning the data available in the Polish SDI are reported. “SDI usefulness for environmental resource management” section describes the findings about SDI usefulness from the perspective of the planners in terms of preparing spatial plans and tasks concerning environmental conservation and sustainable development.

### The Business Process of the Preparation and Endorsement of a Local Spatial Plan

**Fig. 5:**
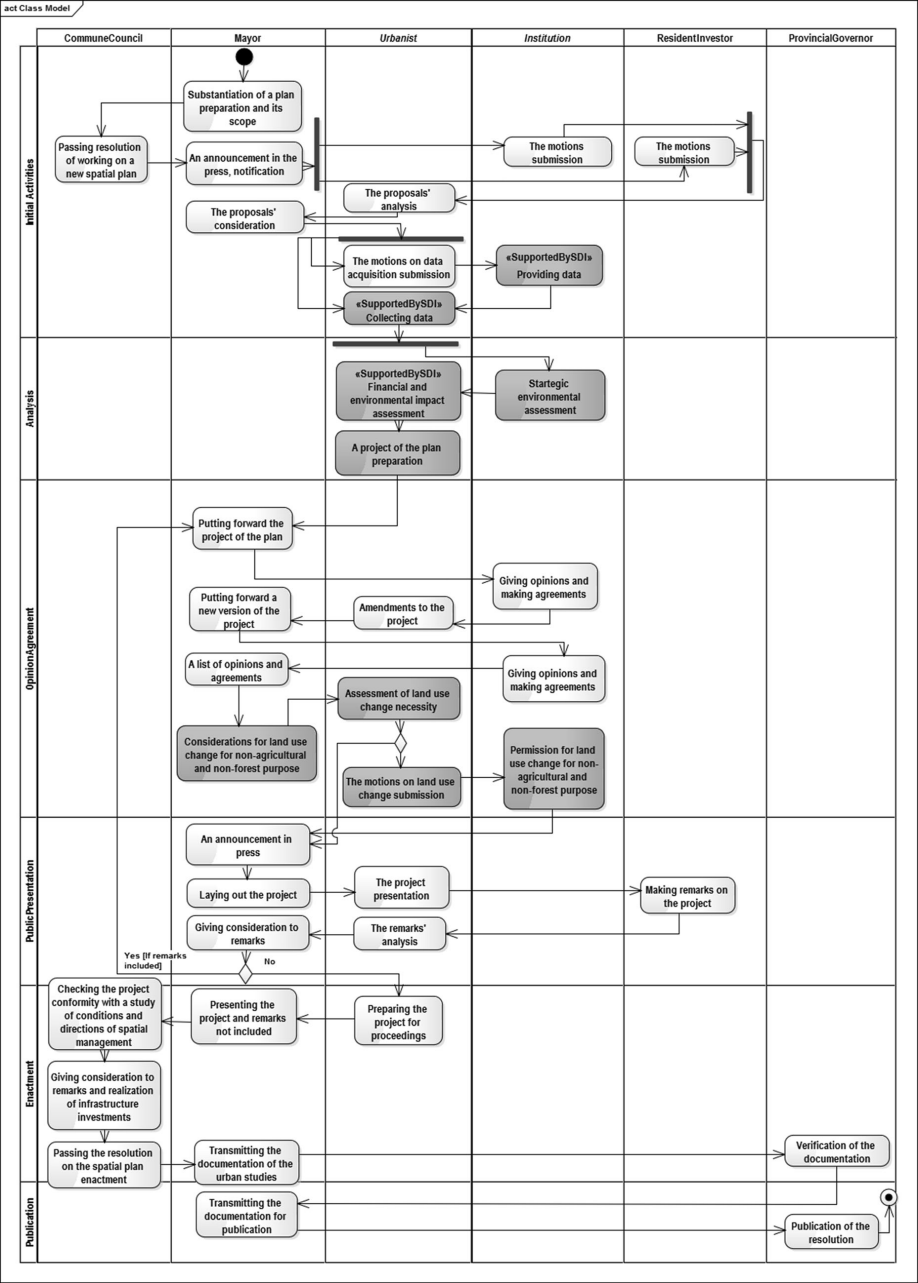
UML activity diagram of the business process of a local plan preparation and endorsement

A business process is a set of one or more linked procedures or activities which collectively realize a business objective or policy goal, normally within the context of an organizational structure defining functional roles and relationships (Workflow Management Coalition 1999). Automated activities are capable of computer automation using a workflow management system to manage the activity during execution of the business process of which it forms a part. Figure 5 illustrates the business process of the preparation and endorsement of a local spatial plan. For automated activities, which are supported by the SDI, the stereotype *SupportedBySDI* is used.

There are six major participants involved in the preparation and endorsement of a plan and six phases of the process. The participants are tasked with submitting motions, sharing data, giving opinions and agreements, verifying documentation, passing resolutions, preparing the final versions of plans, and endorsing spatial plans.

Taking environmental issues into account, the activities listed below (Fig. 5, dark gray) are subject to a more detailed analysis. Three activities are carried out by institutions, one by mayors, and five by urban planners.

*Providing data* are the task of the institutions which have been made responsible by law for datasets and registers and which establish rules and information technology for data access and use. Information systems and infrastructures are maintained and developed in accordance with the Regulation on the National Interoperability Framework, the Minimum Requirements for Public Registries and Electronic Information Exchange as well as the Minimum Requirements for IT Systems (Polish Parliament 2012b). Spatial data in the SDI are in the electronic format and are required to conform to the implementation rules and regulations. However, in the case of information systems implemented by certain authorities and data providers, considerable effort is to be put into coordinating the management of the system, capturing data in the electronic format and defining data models.

*Strategic environmental impact assessment* is defined in the Act On Environmental Information Sharing and Its Protection, Public Participation In Environmental Protection and the Environmental Impact Assessment (Polish Parliament 2013a), and it refers to the procedure for the assessment of the impact of local plans on the environment. The procedure consists of four stages as follows: agreement on the scope of information included in the environmental impact assessment documentation, environmental impact assessment, approval of the documentation, and public participation.

*Permission for land*-*use change for nonagricultural and nonforest purpose* is issued solely in local plans pursuant to the Act on Protection of Agricultural and Forest Land (Polish Parliament 2013c). Arable lands in class I–III and state-owned forest areas require the permission of the Minister of Agriculture and Rural Development and the opinion of the province marshal. In the case of other forest areas, the province marshal is the authority giving permission after receiving the opinion of the Chamber of Agriculture. In the case of state-owned forest areas, the opinion of the Director of the Regional State Forest Directorate is mandatory. If a given forest area constitutes the property of a national park, the director of that park is to give their permission.

The activity of *Considerations for land*-*use change for nonagricultural and nonforest purpose* applies to mayors and is followed by the assessment of the land-use change necessity made by the urban planner.

In the *Assessment of land*-*use change necessity*, the assumption is that the land-use change concerns only badlands, yet a detailed analysis of the social, economic, and environmental conditions is recommended.

*Collecting data* is often the most time-consuming activity, yet of great importance to the task of *Preparation of the project of a spatial plan*, and it includes not only data acquisition but also gathering information about data sources and providers. A parallel activity is the submission of motions regarding data acquisition. After obtaining access to data, they are verified against the needs of the planner.

The scope of the *Financial and environmental impact assessment* is defined in the Regulation on the Required Scope of the Project of a Local Development Plan (Polish Parliament 2003). The assessment includes the impact of a local plan on the earnings and expenses within the commune and also on the expenses related to the execution of technical infrastructure investments. Supplementary parts of the documentation comprise motions and recommendations on the planned land use. The activity is preceded by the agreement on the scope of information included in the documentation of environmental impact assessment.

*The motions on land*-*use change submission* are prepared when the decision on the change of land use is made by the urban planner. The mandatory part of the motion includes a justification for the decision, a list of areas, and an economic justification for the planned land use. A map of the commune or city with build-up areas constitutes an attachment to the motion.

A further study of spatial planning activities performed by urban planners as well as environmental resource management issues is presented in Sect. 5.2.

Table 2 demonstrates a list of twenty-three institutions involved in environmental management, which take part in the preparation of local plans and the process of providing opinions and reaching agreements. The Act on Spatial Planning and Land Development (Polish Parliament 2015) indicates nine participants in the procedure. Apart from the aforementioned Act, on the basis of four additional regulations, the group of the involved institutions and authorities is increased by fourteen.

**Table 2:** Institutions involved in environmental management which participate in the procedure of preparation of local plans in Poland

No.	Legal basis	Institution	Type of participation	Environmental issues subject to opinion/agreement
1.	Act on spatial planning and land development (Polish Parliament, 2015) (total number of participating institutions: 9)	Regional director for environmental protection	Opinion (mandatory)	The whole project of a plan
		Province geologist, district geologist	Opinion (optional)	Underground bodies of water, mineral deposits
		Fire service	Opinion (optional)	Locations for new factories facing failure risk
		Province Inspector of Environmental Protection	Opinion (optional)	
		Province sanitary inspector	Opinion (optional)	The whole project of a plan
		District governor	Opinion (optional)	Areas facing landslide risk
		Mining authority	Agreement (optional)	Development of mining areas
		Minister of health	Agreement (optional)	Development of health resort conservation areas
2.	Act on water management (Polish Parliament, 2012a) (total number of participating institutions: 1)	Regional Water Management Authority	Agreement (optional)	Development of conservation areas for water intakes, conservation areas for inland bodies of water and areas facing flood risk
3.	Act on environmental information sharing and its protection, public participation in environmental protection, and the environmental impact assessment (Polish Parliament, 2013a) (total number of participating institutions: 2)	Regional director for environmental protection	Opinion (mandatory)	Assessment of environmental impact
		District Sanitary Inspector	Opinion (mandatory)	
4.	Act on nature protection (Polish parliament, 2013b) (total number of participating institutions: 1)	Regional director for environmental Protection	Agreement (optional)	National park, nature reserve, landscape park, protected area, existing and planned Natura 2000 sites
5.	Act on protection of agricultural and Forest Land (Polish Parliament, 2013c) (total number of participating institutions: 5)	Minister of Agriculture and Rural Development	Agreement on land-use change for nonagricultural and nonforest purpose (optional)	Arable lands in class I-III
		Minister of the environment		State-owned forest lands
		province marshal		other forest lands
		Director of the regional state forest directorate	Opinion in the course of gaining approval for land-use change for nonagricultural and nonforest purpose (optional)	State-owned forest lands
		director of a national park		Lands of national parks
6.	Good practice (total number of participating institutions: 5)	Utility network management unit	Information (optional)	Existing and planned utility networks
		Forest Inspectorate	Information (optional)	Forest lands

### Local Plans and Environmental Resource Management

Spatial planning in the context of environmental management signifies the inclusion of environmental indicators in the process of future land-use planning as well as the measurement of the possible environmental impact exerted by the planned land use depicted in local plans. The decisions of the urban planner about the possible utilization of the land in the future need to support nature conservation and sustainable development.

#### Inclusion of Environmental Indicators in Spatial Plans

Planning future land use depends on the environmental conditions in the location covered by the local plan, which determine the course of the process. First, the identification of the environmental elements, e.g., energy and natural resources, water, land use, population and housing, agricultural crops, and transportation is performed.

Furthermore, the following boundary lines and land-use zoning elements of the biological diversity or environmental protection are included in the local plan:

- Mining regions and areas,
- Documented underground bodies of water with planned water intake conservation areas and areas of conservation of underground bodies of water,
- Areas of conservation of water intakes and inland bodies of water,
- Maps of flooded areas and risk maps,
- Areas particularly exposed to flood risk,
- Areas facing landslide risk,
- Boundary lines of conservation areas,
- Air, noise, water and soil, vibration, and electromagnetic field protection areas,
- Forest management plans,
- Zones of land-use change for nonagricultural and nonforest purpose,
- Health resort conservation areas,
- Documented carbon dioxide underground storages.

The next step is spatial design, which relies on the results of the environmental analysis, for the purpose of defining the following five indicators:

- Minimal and maximal density of buildings,
- Minimal percentage of vegetation areas and bodies of water,
- Maximal height of buildings,
- Building lines,
- Size of buildings.

#### Supporting Nature Conservation and Environmental Management

After evaluating environmental elements identified for the area defined by the boundaries of a local plan, the planner begins working on future land use, a task of great importance and responsibility, which has an impact on the quality of space and environmental management. Among other things, the local plan establishes the following:

- Rules for water management, sewage disposal, waste management, and transportation infrastructure,
- Management restriction zones, industrial, and manufacturing areas,
- Noise protection zones surrounding urban areas and within urban areas,
- Rules for conserving bodies of water and flood protection,
- Rules for managing bodies of water, developing conservation areas for water intakes and inland bodies of water,
- Bans on enclosing properties adjacent to public inland bodies of water,
- Directions for utility network development.

The tasks of urban planners involve preventing environmental devastation. For this purpose, the definition of special conditions of land development and restrictions on land use are applied. The tightest restrictions affect areas with a ban on building-up. Ecological restoration is achieved by including areas which require environmental rehabilitation in local plans.

Pro-ecological activities relate to planning built-up areas for renewable energy sources. This shows that pro-ecological policy has a significant impact on the natural landscape. Notably, the challenging part in this case is the lack of an objective assessment of landscape values.

Local plans define the conditions for executing investments and thereby support pro-effective activities to the benefit of nature conservation.

### Data Sources in the National Spatial Data Infrastructure

This section focuses on data availability for the purpose of preparing spatial plans and managing environmental sources from the viewpoint of urban planners.

#### Data Available Through the Polish Access Point

Data availability depends upon the content and completeness of datasets on a variety of spatial themes (Nedović-Budić et al. 2004). The data characteristics for the measurement of data availability in this study are (Zwirowicz-Rutkowska 2015): (1) thematic accuracy; (2) completeness; (3) spatial resolution; (4) temporal validity; (5) positional accuracy; (6) distribution format; and (7) lineage.

**Fig. 6:**
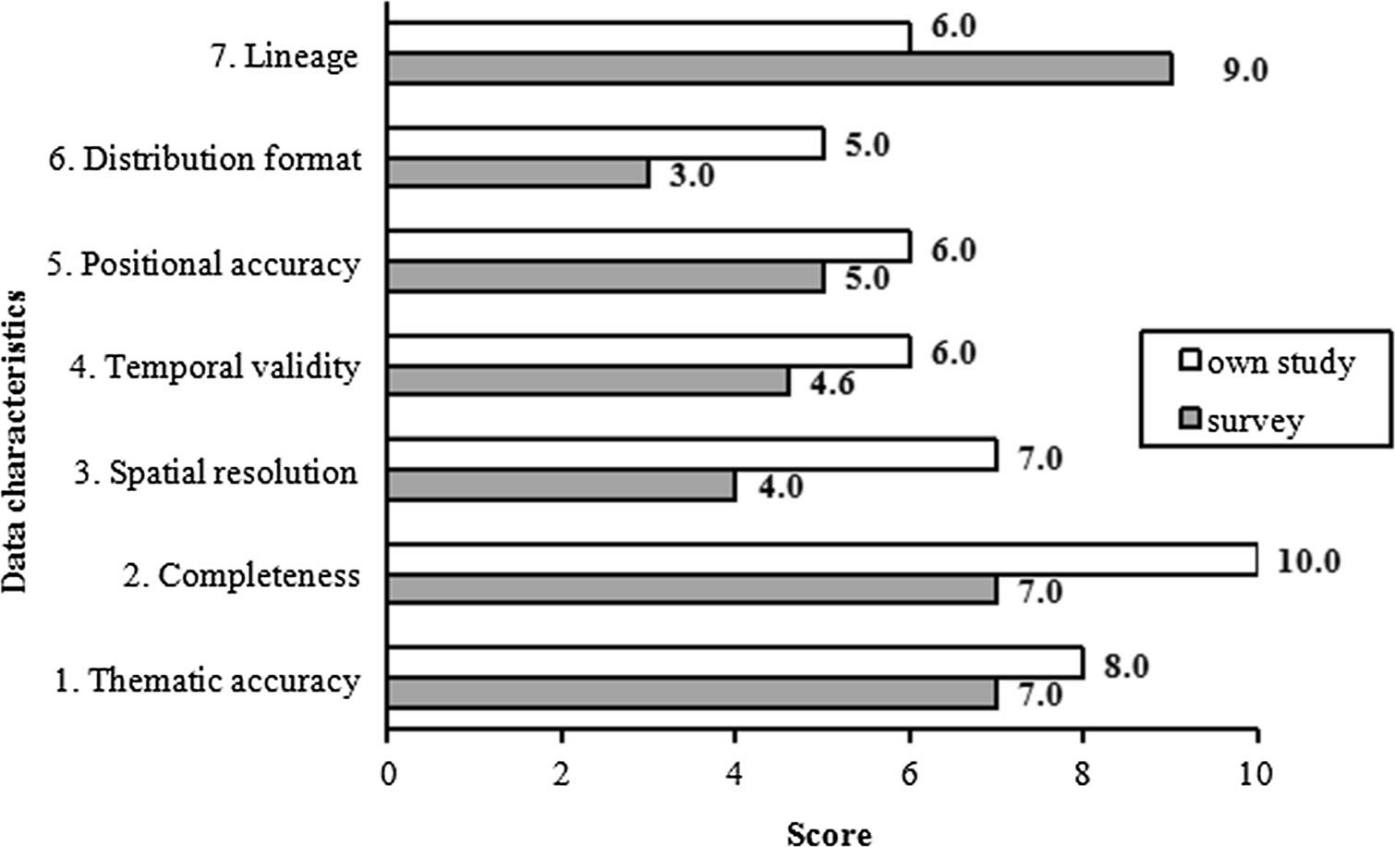
Assessment of data available through the Polish access point

Figure 6 presents a summary of the results of the data availability assessment which was conducted in two ways. The results of the survey conducted in 2014 (Fig. 6 indicated in *gray*, Zwirowicz-Rutkowska 2015) show that, on the one hand, planners value data for their lineage (an average score of 9) but, on the other hand, they emphasize the need for proper distribution formats (an average score of 3 for Distribution format indicator). The total data availability score was 5.7. The results of the authors’ own study conducted in 2015, despite assigning higher values for six out of seven indicators, confirmed the distribution format issue, but depreciated the attribute of data lineage.

Out of forty-two web services and geoportals analyzed in this study, seventeen are integrated into the national access point (Table 3). Over 88 % of them have a national range. These geoportals and websites are the source of 88 data layers. Adhering to the INSPIRE Directive (European Parliament and the Council 2007), the data used by planners are mostly related to fifteen spatial data themes, including nine from Annex III, three from Annex I, and the rest from Annex II.

**Table 3:** The most important data sources are websites and geoportals accessed through the national access point (Accessed December 2014)

No.	Addresses of websites and geoportals	No. of layers	National range	Local/ regional range	INSPIRE spatial data theme
1.	http://www.gismazowsza.pl/	5	0	1	3.4. Land use
2.	http://mapy.geoportal.gov.pl/imap	2	1	0	1.6. Cadastral parcels 3.2. Buildings
3.	BDOT—http://mapy.geoportal.gov.pl/wss/service/WMTS/guest/wmts/TBD BDO - http://mapy.geoportal.gov.pl/wss/service/WMTS/guest/wmts/BDO VMAP—http://mapy.geoportal.gov.pl/wss/service/WMTS/guest/wmts/VMAP	27	1	0	3.2. Buildings
4.	http://mapy.geoportal.gov.pl/wss/service/wmspub/guest/AK_7_Mapa_proponowanych_kierunkow_zagospodarowania/MapServer/WMSServer	1	1	0	2.2. Land cover 2.4. Geology
5.	http://mapy.geoportal.gov.pl/wss/service/pub/guest/G2_TRANSPORT_WMS/MapServer/WMSServer http://mapy.geoportal.gov.pl/wss/service/INSPIRE_TN_TBD/guest	1	1	0	3.6. Utility and governmental services
6.	http://mapy.geoportal.gov.pl/wss/service/img/guest/Krajobrazowa/MapServer/WMSServer	1	1	0	2.1. Elevation
7.	http://mapy.geoportal.gov.pl/wss/service/img/guest/CIEN/MapServer/WMSServer	1	1	0	
8.	http://mapy.geoportal.gov.pl/wss/service/img/guest/HIPSO/MapServer/WMSServer	1	1	0	
9.	http://mapy.geoportal.gov.pl/wss/service/wmsimg/guest/ISOK_HipsoDyn/ImageServer/WMSServer	1	1	0	
10.	http://geoserwis.gdos.gov.pl/mapy/	15	1	0	1.9. Protected sites 3.18. Habitats and biotopes 3.19. Species distribution
11.	http://www.bdl.lasy.gov.pl/portal/mapy	15	1	0	2.2. Land cover (partly)
12.	http://geoportal.pgi.gov.pl/portal/page/portal/SOPO/Wyszukaj3	2	1	0	2.4.Geology 3.12. Natural risk zones 3.20. Energy resources 3.21. Mineral resources
13.	http://mapy.geoportal.gov.pl/wss/service/img/guest/HYDRO/MapServer/WMSServer	1	1	0	1.8. Hydrography 3.11. Area management/restriction/regulation zones and reporting units
14.	http://spdpsh.pgi.gov.pl/GeoPSHv7/ObszZagrPodt/wms	1	1	0	
15.	http://wezelsdi.geoportal.gov.pl/gornawisla/wmservice.aspx	2	0	1	
16.	http://mapy.geoportal.gov.pl/wss/service/wmspub/guest/AK_4b_Mapa_ter_zagr_halasem_Ldwn/MapServer/WMSServer	6	1	0	outside the scope of the INSPIRE
17.	http://mapy.geoportal.gov.pl/wss/service/wmspub/guest/AK_5a_Mapa_rozm_ludnosci_eksponowanej_na_halas_Ldwn/MapServer/WMSServer	6	1	0	
TOTAL		88	15	2	

#### Other Data Sources

The addresses presented in Table 4 have been collected since 2010 during the realization of various local plans and urban studies and they have been additionally verified in 2015 through interviews and surveys directed to the institutions listed in Table 2. Due to the fact that 60 % of all the geoportals and websites analyzed in this study are not accessible through the national access point and are spread across the Internet, it was necessary to check, confirm, or acquire the required information (mainly about the addresses of the websites and the available data formats) directly from the data providers and the authorities responsible for different thematic data and registries.

**Table 4:** Other most significant data sources are thematic websites and geoportals (Accessed May 2010-December 2014)

No.	Addresses of websites and geoportals	No. of layers	National range	Local/ regional range	INSPIRE spatial data theme
1	http://powietrze.gios.gov.pl/gios/	3	1	0	3.7. Environmental monitoring facilities
2	http://mjwp.gios.gov.pl/mapa/	1	1	0	
3	http://inspire.gios.gov.pl/portal/	21	1	0	
4	http://mapy.gios.gov.pl/prtr/	4	1	0	
5	http://www.gis-mokradla.info/	1	1	0	2.2. Land cover
6	http://crfop.gdos.gov.pl	1	1	0	1.9. Hydrography 3.18. Habitats and biotopes 3.19. Species distribution
7	http://mapa.ekoportal.pl/	14	1	0	
8	http://www.ibs.bialowieza.pl/	1	1	0	
9	np. http://rdlpol.gis-net.pl/ http://mapa.poznan.lasy.gov.pl/ http://mapa.radom.lasy.gov.pl/ http://mapa.katowice.lasy.gov.pl/	33	0	1	2.2. Land cover (partly)
10	https://www.ibles.pl/mapa/index.html	13	1	0	
11	http://epsh.pgi.gov.pl/epsh/	11	1	0	2.4.Geology 3.12. Natural risk zones 3.20. Energy resources 3.21. Mineral resources
12	downloadmanager@pgi.gov.pl	20	1	0	
13	http://geoportal.pgi.gov.pl/portal/page/portal/MIDASGIS/TabZloza:search	1	1	0	
14	http://web3.pgi.gov.pl/website/cbdg/viewer.htm	24	1	0	
15	http://geozagrozenia.pgi.gov.pl/	1	1	0	
16	http://mapy.isok.gov.pl/imap	3	1	0	
17	http://www.smorp.pl/imap/	18	0	1	1.8. Hydrography 3.11. Area management/restriction/regulation zones and reporting units
18	http://geoportal.kzgw.gov.pl/imap/	16	1	0	
19	http://geoportal.kzgw.gov.pl/services/KZGW_2012/13Obiekty_Pietrzace_WMS/MapServer/WMSServer	1	1	0	
20	http://geoportal.kzgw.gov.pl/services/KZGW_2012/5MPHP2010/MapServer/WMSServer	1	1	0	
21	http://geoportal.kzgw.gov.pl/services/KZGW_2012/3JCWP/MapServer/WMSServer	1	1	0	
22	http://geoportal.kzgw.gov.pl/services/KZGW_2012/4JCWPd/MapServer/WMSServer	1	1	0	
23	http://geoportal.kzgw.gov.pl/services/KZGW_2012/1ObszaryDorzeczy/MapServer/WMSServer	1	1	0	
24	http://mapy.geomelioportal.pl/	82	0	1	3.9 Agricultural and aquaculture facilities
25	http://modulsdi.geoportal.gov.pl/sdiportalprg/	12	1	0	3.16. Sea regions
TOTAL		285	22	3	

Over 88 % of the data sources have a national range. Data used by urban planners are mostly related to (European Parliament and the Council 2007), thirteen spatial data themes, including nine from Annex III, two from Annex I, and the rest from Annex II.

**Fig. 7:**
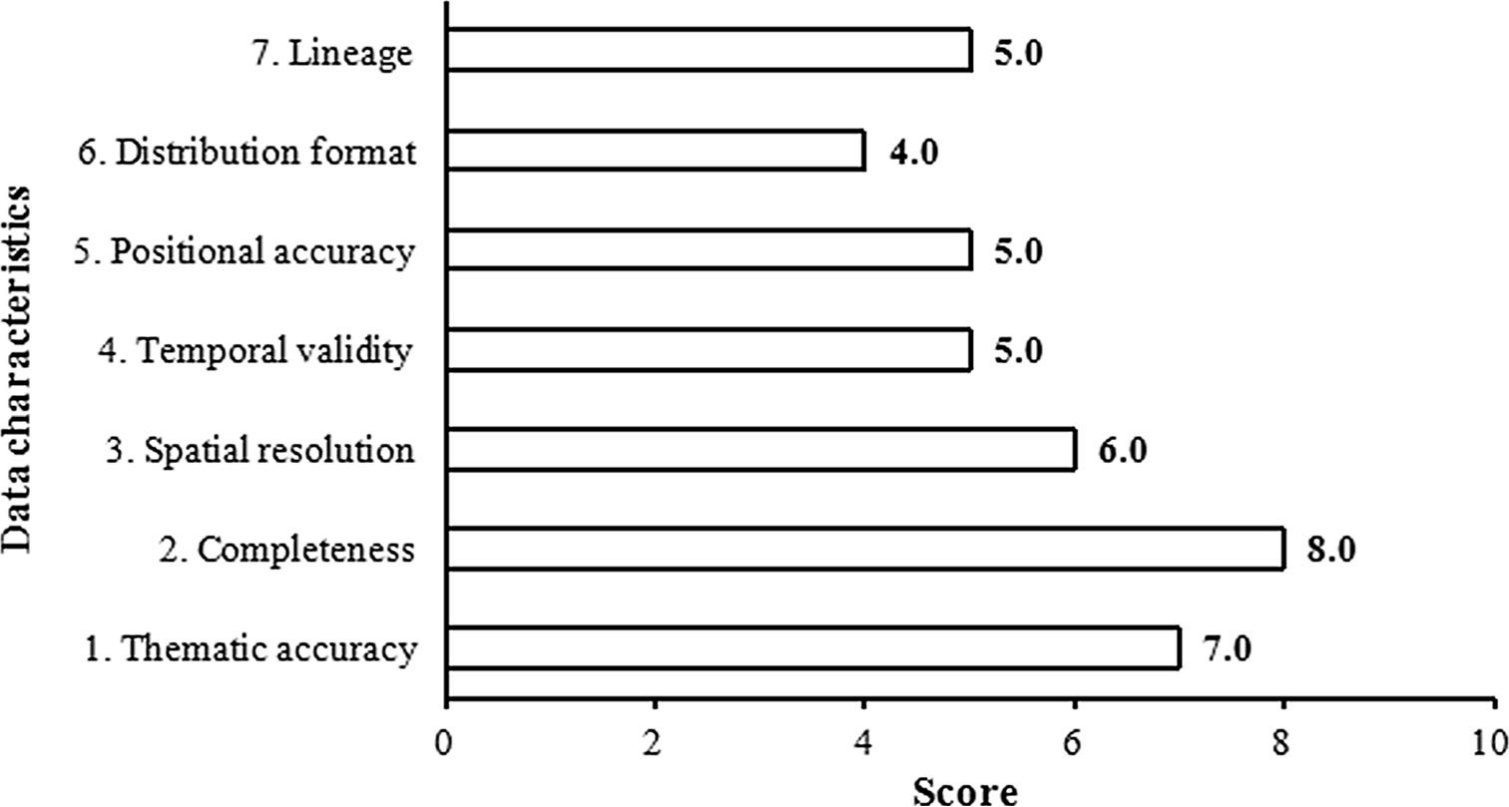
Assessment of data available through other websites and geoportals

Figure 7 presents average scores of data availability. On comparison of the results of the assessment with the scores for data sources available through the national access point, it can be noted that the latter is better-rated. Data sources integrated into the national access point are more complete.

## SDI Usefulness for Environmental Resource Management

As mentioned in Sect. 5.1, the preparation of local plans is supported by the SDI, especially in such activities as (Fig. 5) *Providing data* by institutions, *Collecting data*, and also *Financial and environmental impact assessment* performed by planners. This section describes the results of the assessment of the National Spatial Data Infrastructure usefulness and the Geoportal 2 map viewer usability for the tasks of urban designers with regard to the inclusion of environmental indicators in spatial plans and decision-making processes that create environmental impact.

### Usability of the Polish Access Point

In this study, usability is described by (Zwirowicz-Rutkowska 2015) the level of functions intuitiveness, clarity, and content presentation of the map viewer.

**Fig. 8:**
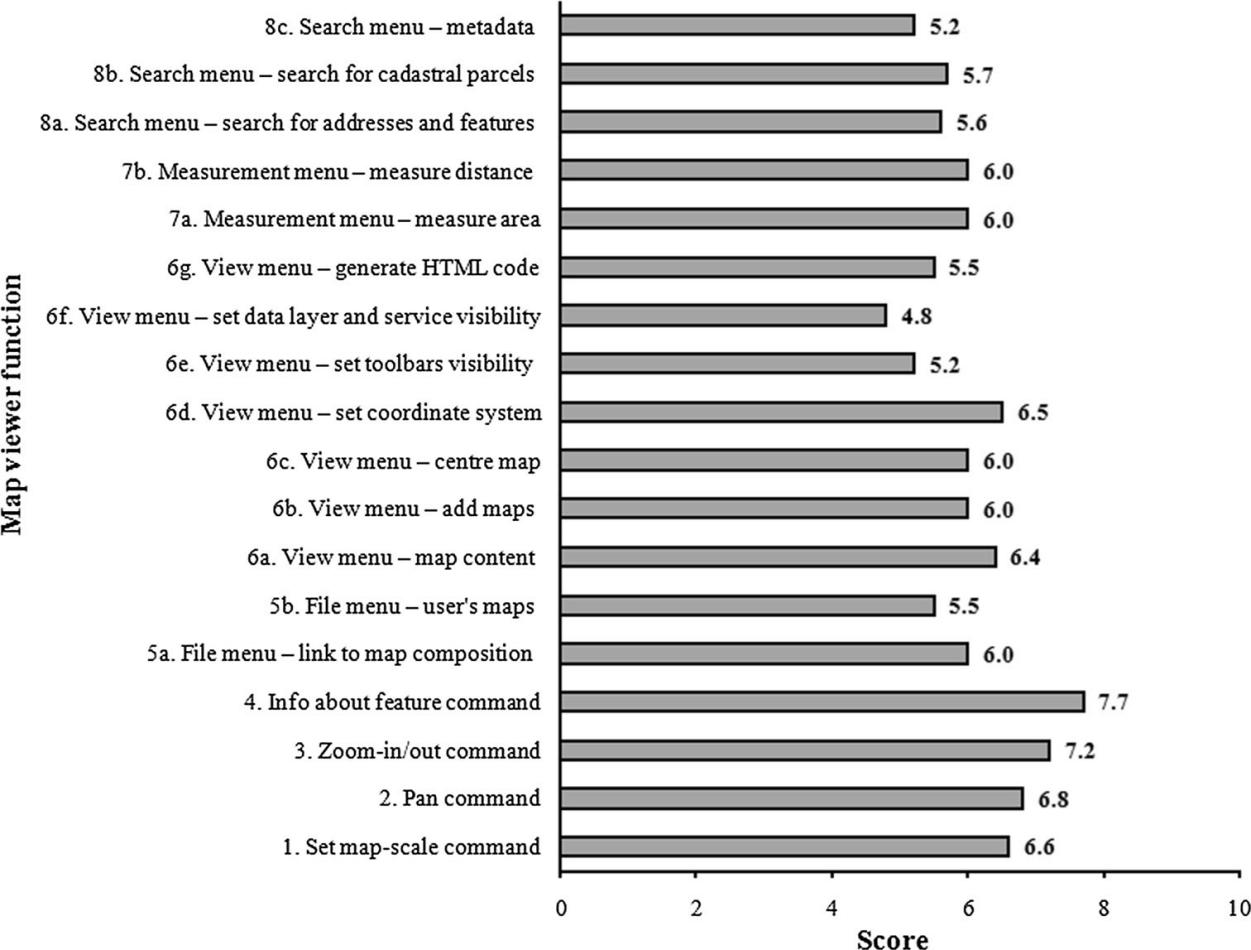
Assessment of the Geoportal 2 map viewer functions

The average score of the Geoportal 2 map viewer usability was 6.0. Figure 8 shows detailed information about eighteen features and functions of the map viewer presented in Table 1. Only two commands, i.e., *Info about feature* and *Zoom in/out* received a score above 7. The other nine functions, which are connected with measures, panning, setting the map scale, and also partly view commands, seemed to be sufficiently usable (scores between 6 and 6.8) for urban planners who have declared their use of this map viewer’s functionality. The commands of the search menu, three functions of the view menu as well as the operation of the file menu, i.e., *User’s maps,* are perceived to be less user-friendly.

The same survey results also show that out of eighteen features and functions of the map viewer, nine (i.e., 5a–6g, Table 1) are either unknown or known but have not yet been used in spatial planning tasks for almost 48 % of the respondents.

### Usefulness of the Polish Access Point

Usefulness means that a system’s functionality actually makes sense and adds value in relation to a particular work setting (Blomberg et al. 1994). The list of the potential SDI use categories in the area of spatial planning practice includes (Zwirowicz-Rutkowska, 2015) as follows: (1) agreements; (2) factors and land-use trend analysis; (3) production of a comparative variant study; (4) field studies; and (5) other. Considering the functions for reading, visualizing, and analyzing spatial data, the following five levels of use can be distinguished: (1) use of the geoportal map viewer; (2) connecting to web servers in the GIS/CAD software; (3) viewing maps; (4) displaying data from different sources (the geoportal map viewer and/or GIS/CAD software); and (5) data manipulation and transformation.

The study revealed that the use of the SDI in the area of spatial planning practice mainly includes field studies, analyses of factors, and analyses of land-use trends. Reading, visualizing, and analysis of spatial data chiefly focus on viewing maps in the map viewer and displaying data from different sources, thus not on the use of the GIS/CAD software.

Taking into account the usefulness of the applications of the main access point, the national geoportal and the statistics module are considered the most valuable for urban planners.

### Impact of the SDI on Decision-Making Processes in the Area of Environmental Management

**Fig. 9:**
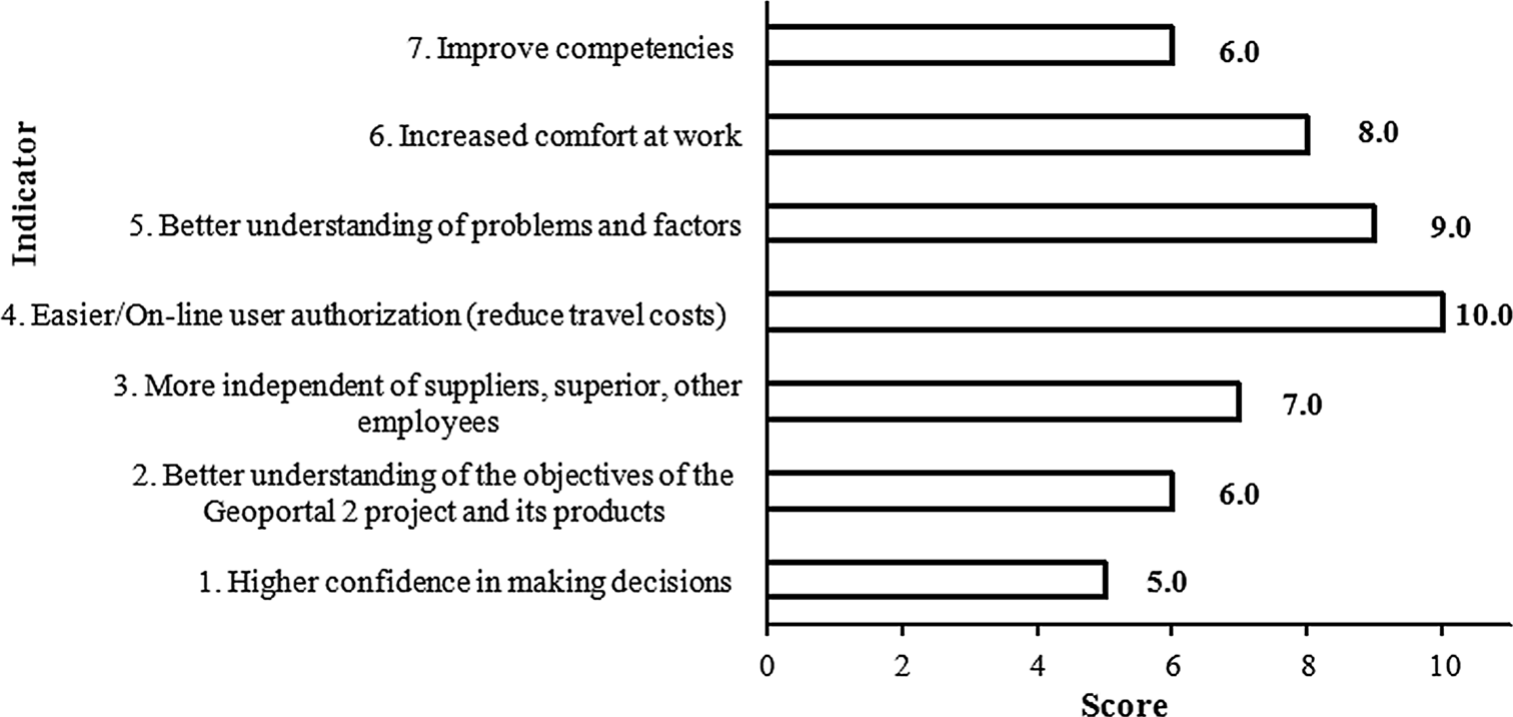
Impact of the Polish SDI on decision makers in the area of environmental management

**Fig. 10:**
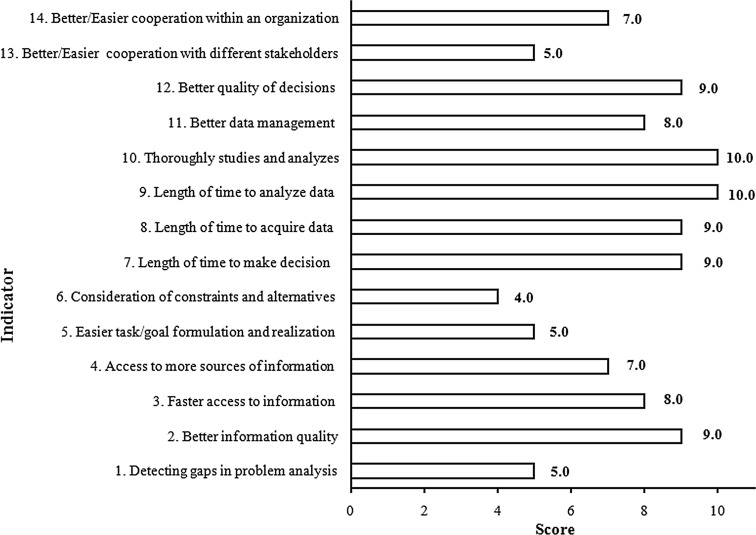
Impact of the Polish SDI decision-making processes in the area of environmental management

The expert knowledge gained by the authors through the study of the impact of the Polish SDI on decision makers and decision-making processes in the area of environmental management resulted in specific findings presented in Figs. 9, 10.

One of the most important benefits for decision makers (Fig. 9) is easier authorization to receive or download data and a reduction in travel costs (a score of 10). The national spatial data infrastructure improves the understanding of environmental factors during urban designing (a score of 9) and increased comfort at work (a score of 8) resulting from the possibility of gathering many different sources of data.

The whole process of decision making (Fig. 10) seems to take less time due to efficiency improvements in data acquisition and analysis (a score of 10 for indicators no. 9 and 10) as well as better information quality (a score of 9). Also, an increase in the number of broader studies and analyses can be observed, as more thematic data layers from different data providers are able to be displayed at the same time.

## Discussion

The field of environmental management is highly complex when its universe of discourse or the number of both objects and tasks under investigation, as well as correlations between different factors are analyzed. It is also changing as human activity exerts an influence on the environment and the processes and interactions between the environment’s components also affect human economic activity. Moreover, it concerns all three main sectors of socio-economic activity of humans (i.e., public, private and NGOs). What should also be noted is the interdisciplinary character of environmental management issues, which is a result of multidirectional study of phenomena, as well as engagement of different field professionals. Taking all the above mentioned factors into consideration, instruments of any kind, which could support tasks in the area of environmental management, are warmly welcomed. The literature overview presented in this study showed that ICT in general, but also GIS and SDI have become very important tools to support environmental research, learning and decision making. The International Telecommunication Union (ITU) (2008) underlines the role of ICT-oriented applications in environmental protection and the sustainable use of natural resources, as well as forecasting and monitoring the impact of natural and man-made disasters. This paper focused on presenting evidence of SDI usefulness in the area of environmental management if one of the professional groups—spatial planners—engaged in the process of providing sustainable development is taken into account.

The decisions of urban planners about possible land utilization in the future need to support conservation and sustainable development of the natural environment. Spatial plans include many different environmental indicators, but also establish many rules regarding conservation of the natural environment. Figure 5, even though based on a procedure that is standard in Poland, shows some phases (initial activities, analysis, opinion and agreement, public presentation, enactment) which are applicable for many countries (cf. Lotfi 2012; Dutt and Costa 1985). The activities performed with support of the Spatial Data Infrastructure (Fig. 5) by many Polish planners, i.e., collecting data, financial, and environmental impact assessment, as well as by institutions involved in environmental management which participate in local planning (i.e., providing data), could suggest an approach to be taken in order to realize goals concerning environmental issues and spatial planning practices.

However, in order to implement this approach into any SDI, some conditions must be fulfilled. First of all, the objective of the SDI’s assistance in the area of environmental management and spatial planning should be articulated by the organization responsible for the SDI. Consideration of potential and appropriate data sources and sets for this purpose is also essential. Another important issue is agreements and procedures of cooperation between data suppliers, the organization managing the SDI and the users. The suppliers should be responsible for assuring data interoperability with SDI components, e.g., the main data access point and network services (geoportal). Functionalities covering urban planners’ expectations are also an issue of great importance.

Generally, the Polish SDI is valuable because it allows for the acquisition of data on environmental monitoring facilities, agricultural and aquaculture facilities as well as sea regions. Both the main access point and other data sources presented in Tables 3, 4 are useful, although selected websites and geoportals maintained by different data providers but coordinated by the same institution duplicate certain datasets which are of different completeness and temporal validity. In many cases, the authorities of a given level undertake objectives which do not seem to be consulted with the appropriate authorities of other levels, even though the Act on Spatial Information Infrastructure (Polish Parliament 2010) and other regulations define specific coordination procedures and duties. Unfortunately, as the study and the interview results demonstrated, there are cases in which regulations do not translate well into practice.

Only 20 % of data suppliers provide information about the possibility of obtaining data directly from the responsible party. Links to web services and map viewers as well as information about datasets and web-based map services are rarely included on home pages but instead presented on lower-level pages on the websites of certain institutions. The problem with proper data formats suitable for spatial planning analysis, including the lack of download services, is the same as in the case of the national access point.

The listing shown in Table 4 is based on the authors’ own expert knowledge and shows that every urban planner has their own ways of searching for data sources and acquiring information. There exists no official or trade website integrating addresses of thematic websites and geoportals useful for urban design.

The case study presented in this paper demonstrated the positive impact of PSDI on decision-making processes and numerous activities. Unfortunately, there are also certain time-consuming inconveniences resulting from using geoportals, especially with regard to ones not available through the national access point. For download services, proper applications on data acquisition must be submitted. However, the lack of metadata, including information about the responsible party and conditions for applying for access and use is a common problem. It is worth noting that the Open Data paradigm is making inroads into institutions (the Surveying and Cartographic Documentation Center, the Regional Directorate for Environmental Protection). Practice shows that Open Data reduces the amount of time needed for initial activities which are a part of the decision-making process and make it possible to focus on performing analyses and data manipulation. The Open Data Catalogue of the Regional Directorate for Environmental Protection provides data on conservation categories and areas as well as Ramsar sites. It is also worth emphasizing that these are vector data and thus more advanced data manipulation and transformation can be performed.

There are several conclusions that can be drawn from the Polish case study. The kinds of applications and functionality that could be offered by the SDI were presented. The case study also demonstrated the importance of effective cooperation between data providers and organizations responsible for the SDI. Many procedures, including data acquisition and payment should not follow the traditional bureaucratic route, but should be adjusted to solutions offered by e-government systems, since the SDI should be perceived as their integral component. The SDI potential lies not only in the main access point but also in other integrated thematic infrastructures, although the main access point should be an efficient resource broker and information point for the entire SDI. Usability monitoring, as well as constant thematic and technical development of the SDI is critical for maintaining its usefulness as well as the good image of public authorities and their information infrastructure.

SDI design and implementation requires establishing principles and making decisions regarding datasets, standards, software and hardware, procedures, networking, financial resources, and many more. Another key aspect is the definition of SDI objectives and communities interested in the development of such infrastructure. As the datasets could be made available through network services, the challenge for SDI administrators is to ensure that server performance and scale will be maintained at appropriate levels in order to keep up with users’ computing demands. Maintaining the technical integration of nodes in the main access point as well as data interoperability and updating are also a part of the challenge. It should also be noted that data accessibility (i.e., discovery and viewing services) depends on, among others, the data providers’ server status and, in the event some of them are disabled, some data sources would become unavailable to users. Other factors which influence the quality of SDI use include Internet connection quality on both sides, as well as user hardware and software. Since environmental management includes many fields of concern, including biological conservation, natural resource management, environmental economics, and sustainable development (cf. e.g., Dolný and Harabiš 2012; Tear et al. 2014; Nungesser et al. 2015; Alberdi et al. 2016), as well as organizations, groups and professions engaged in the process (cf. e.g., Hosseini and Rezaei 2013; de Nooy 2013; Qu et al. 2014; Henckens et al. 2016; Romañach et al. 2016), it is very difficult to develop one application or access point which would satisfy needs and expectations of all applicable groups of users, taking into account functionality, available datasets or conditions of data use. Taking network services required for operation and maintenance of the SDI into account, the map viewer functionalities presented in this study (Table 1) and suggestions of spatial planners concerning data formats allow for a type of universal solution to be suggested. At the same time, it is also possible to consider the development of more dedicated applications or subthematic infrastructures.

Nevertheless, the importance of the SDI in environmental management is noticeable and could be considered on several levels. On the data level, the SDI offers cohesive datasets which could be viewed directly via the SDI interfaces, e.g., through a geoportal and also using different software solutions that provide connection to WMS/WFS/WCS servers, making possible a wide range of operations and data analyses. The SDI ensures better information quality and provides an advantage of operating on more thematic data layers based on information from different data providers which could be displayed at the same time. From the perspective of the communities involved in enacting policies and making decisions pertaining to environmental issues, the SDI improves the understanding of environmental factors, supplements knowledge with additional conditions, factors and restrictions as well as provides support for environmental studies and analyses with a greater scope, which in turn could lead to more intersectional planning, conflict identification and resolution, sustainable development, monitoring and preservation of biodiversity, as well as environmental resource management. On the data providers’ level, the SDI contributes to the organization of datasets and their popularization among users as well as organizations interested in or participating in environmental monitoring activities. On this level, the SDI also ensures identification of incoherent and duplicated data.

## Conclusions

The paper presents some initial findings on usefulness of the SDI in Poland. The work adds to a growing body of literature on the SDI in the field of environmental management by presenting the results of an ex-post evaluation of the use of the Polish SDI by urban planners in their tasks related to management of environmental sources, as well as discussion on the significance of spatial data infrastructure in environmental management from a user standpoint.

In Poland, the use of SDI in urban studies is connected with the popularization of geographical information systems among planners. Popularization of GIS software in spatial planning, as well as the development of the infrastructure by data suppliers, inclusion of new datasets and the provision of appropriate data formats, might result in a more in-depth use of the SDI and more effective urban design.

The study showed that not all thematic websites and geoportals important to the planners’ tasks can be accessed through the national access point. Still, it should not be overlooked that SDI implementation is, at the time of writing, still a work in progress. However, the results of the investigation presented in this paper prove that there is a noticeable growing trend in using the SDI in preparation of local plans and tasks concerning environmental conservation and sustainable development. The SDI is valuable because it allows for gathering data on environmental monitoring facilities, agricultural and aquaculture facilities as well as sea regions. It also has a positive impact on decision-making processes and improves multiple planners’ activities concerning both inclusion of environmental indicators in spatial plans and support for nature conservation and environmental management in tasks related to future land use.

The results presented in this study have shown that the INSPIRE-mandated national geoportal, which is integrated into the European Community geoportal operated by the European Commission, is not the main web application used for tasks related to urban design. Urban planners would rather use the national access point, the statistics module, as well as over 20 different thematic websites and geoportals instead.

Although the Polish SDI solves certain problems caused by the incompatibility of data formats, low accessibility as well as gaps and inconsistencies in the data, based on the results of the study presented in Sects. 5 and 6, specific improvements to SDI development might be proposed in order to benefit environmental management and spatial planning. First of all, developing an intuitive national geoportal and adding new functionality to the SDI applications would certainly improve the effectiveness of the urban design process. Additionally, the inclusion of metadata in the national geoportal concerning data providers and their geoportals, coordinated by public authorities, whether or not accessed through the national access point, may be suggested, for example on a separate lower level web page. In the case of the latter, a data supplier would provide information about their websites to the administrator of the national geoportal and after verification, the administrator would make the metadata available on the Geoportal 2 website. Moreover, integration of certain e-services and the e-government paradigm (e.g., web services for handling orders and payments) with geoportals and websites of data providers would undoubtedly reduce the time needed for the preliminary activities connected with applications regarding data acquisition, preparation and submission. From the standpoint of urban planners’ needs, the usability of an access point depends on the availability of certain functions, i.e., comprehensive search menus or adding custom maps, necessary for carrying out spatial planning tasks.

Better coordination of operations between different departments of the same institution, as well as enhanced cooperation between different data providers coordinated by one institution, is recommended in order to facilitate the exchange of information and improve the quality of services provided. In addition, it is essential to promote the SDI and to encourage its use by both urban planners and new groups of potential users. Various tutorials, videos, or user guides would provide support for users with little or no experience in the area of IT, the GIS solution, SDI and outputs of the Geoportal 2 project in particular, in order to help them learn the basics and maximize the added value of the solutions.

The sample size and the number of professional groups considered in this study allowed only for a preliminary assessment of the PSDI usability in the area of managing environmental issues. As environmental management is within the range of interest of different professional groups, further studies are recommended in order to broaden knowledge about the usefulness and role of the SDI, especially when considered from different perspectives.
